# UCH-L1 and GFAP: Efficient biomarkers for diagnosing traumatic brain injury

**DOI:** 10.1016/j.bas.2024.102913

**Published:** 2024-08-06

**Authors:** Thangavel Lakshmipriya, Subash C.B. Gopinath

**Affiliations:** Center for Global Health Research, Saveetha Medical College & Hospital, Saveetha Institute of Medical and Technical Sciences (SIMATS), Thandalam, Chennai, 602105, Tamil Nadu, India; Center for Global Health Research, Saveetha Medical College & Hospital, Saveetha Institute of Medical and Technical Sciences (SIMATS), Thandalam, Chennai, 602105, Tamil Nadu, India; Faculty of Chemical Engineering & Technology, Universiti Malaysia Perlis (UniMAP), 02600, Arau, Perlis, Malaysia; Institute of Nano Electronic Engineering, Universiti Malaysia Perlis (UniMAP), 01000, Kangar, Perlis, Malaysia; Department of Technical Sciences, Western Caspian University, Baku, AZ 1075, Azerbaijan

**Keywords:** Biomarker, Brain injury, Nanotechnology

Traumatic brain injury (TBI) occurs when the brain is damaged by a violent, abrupt, external impact, causing injury to the brain and leading to serious disabilities such as difficulties in movement, communication, and understanding due to changes in brain functionality (Fig. 1). Despite prevention efforts through lifestyle modification, therapeutic management, and accident safety, TBI cases continue to evolve. Currently, the severity of TBI is assessed using the Glasgow Coma Scale (GCS) score and imaging techniques such as computed tomography (CT) scans and magnetic resonance imaging (MRI). However, these identification methods have limitations in terms of cost efficiency and accuracy. Additionally, due to the heterogeneity of TBI, it is highly correlated with numerous complex pathologies, making it difficult to identify and diagnose the level of trauma. Researchers are increasingly focusing on blood-based biomarkers for diagnosing TBI, which can improve the approaches to TBI treatment and reduce diagnostic costs. Biomarkers such as S100B, glial fibrillary acidic protein (GFAP), neurofilament light, and ubiquitin C-terminal hydrolase-L1 (UCHL1) have been identified as efficient markers for TBI diagnosis. Among these, after extensive research, the biomarkers GFAP and UCHL1 have been accepted by the Food and Drug Administration (FDA) for the detection of brain lesions in TBI (Hossain et al., 2024).

**Fig. 1:**
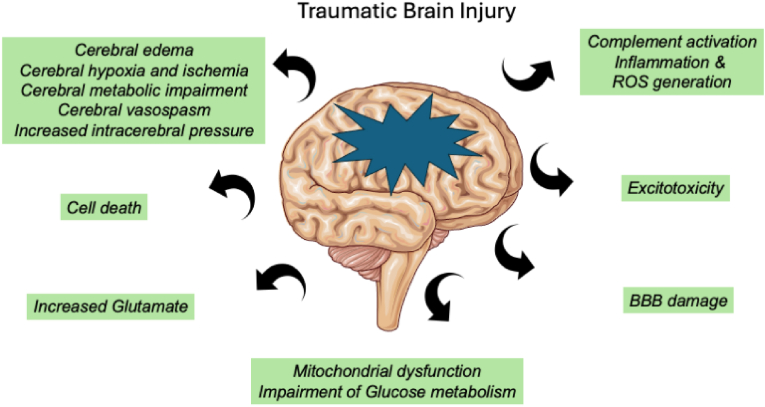
A straightforward diagram showing the pathophysiological reactions that occur after traumatic brain damage and the intricate explosion of subsequent deficits. It should be noted that the blood-brain barrier disruption, neuroinflammation, excitotoxicity, metabolic deficits, apoptosis, oxidative stress, ischemia, and other processes are examples of secondary damage processes associated with traumatic brain injury.

GFAP is a filament protein expressed in the central nervous system (CNS) in astrocytes and is involved in cell movement, structure, and blood-brain barrier function. Recent research has identified that GFAP works as a serum biomarker for TBI, being released after CNS damage. Further research has proven that GFAP levels can be used to identify subtle injury to the CNS and predict the worsening of disability in progressive multiple sclerosis (Lumpkins et al., 2008). Similarly, like GFAP, UCHL1 has been identified as a potential biomarker for TBI. UCHL1 is a neuro-specific protein that is extremely abundant in the brain, estimated to make up 1–5% of the total neuronal protein (Brophy et al., 2011). Research has proven that higher levels of serum UCH-L1 are found in children with brain injuries, and a gradual increase in UCH-L1 levels has been noted across the continuum of TBI severity, from mild to severe (Mondello et al., 2016). Quantifying these two biomarkers helps to diagnose and monitor the condition of TBI.

Various ongoing point-of-care platforms are being developed by researchers to quantify the levels of GFAP and UCH-L1 for the diagnosis of TBI. Different routine analytical techniques, such as PCR and ELISA, are commonly used to quantify TBI biomarkers. However, researchers are still working to develop a highly sensitive and specific biosensor for diagnosing TBI. For instance, a highly sensitive graphene field-effect transistor (GFET) was developed by researchers for the sensitive detection of GFAP in plasma from patients. The anti-GFAP antibodies are conjugated with the GFET device, causing a shift in the Dirac point that correlates with the concentration of GFAP in the plasma. This biosensor effectively detected GFAP at levels as low as 400 aM, enabling the identification of TBI (Xu et al., 2022). In another study, gold nanoparticles functionalized with anti-GFAP antibodies were attached to L-cysteine-modified gold nanoparticles, which were subsequently immobilized on screen-printed carbon electrodes. GFAP was quantified on these surfaces using an impedance electrochemical sensor, lowering the detection limit of GFAP to 51 fg/mL (Ozcelikay et al., 2022). Furthermore, researchers developed electrochemical impedance spectroscopy to quantify UCH-L1 using a transduction method. This system efficiently detects UCH-L1 within 5 minutes across concentrations ranging from 1 to 1000 pM (Lee et al., 2022). Surface plasmon resonance imaging sensors (SPRI) are also employed by researchers for UCH-L1 detection. UCH-L1 is captured from a solution containing anti-UCH-L1 antibodies to form UCH-L1-antibody complexes on the sensing surface. This interaction is monitored by SPRI, detecting UCH-L1 in the range of 0.1–2.5 ng/mL (Sankiewicz et al., 2015). These biomarker quantifications are used to monitor injuries in the central nervous system. Additionally, this sensing system aids in classifying the severity of TBI and improving treatment approaches, thereby reducing diagnostic costs.

## Declaration of competing interest

The authors declare that they have no known competing financial interests or personal relationships that could have appeared to influence the work reported in this paper.
